# Group based diabetes self-management education compared to routine treatment for people with type 2 diabetes mellitus. A systematic review with meta-analysis

**DOI:** 10.1186/1472-6963-12-213

**Published:** 2012-07-23

**Authors:** Aslak Steinsbekk, LisbethØ Rygg, Monde Lisulo, Marit B Rise, Atle Fretheim

**Affiliations:** 1 Department of Public Health and General Practice, Norwegian University of Science and Technology, Post box 8905, Medisinsk teknisk forskningssenter, 7491, Trondheim, Norway; 2 Norwegian Knowledge Centre for the Health Services, Oslo, Norway; 3 Institute of Health and Society, University of Oslo, Oslo, Norway

**Keywords:** Patient education as topic, Self-care, Type 2 diabetes mellitus, Systematic review

## Abstract

**Background:**

Diabetes self-management education (DSME) can be delivered in many forms. Group based DSME is widespread due to being a cheaper method and the added advantages of having patient meet and discuss with each other. assess effects of group-based DSME compared to routine treatment on clinical, lifestyle and psychosocial outcomes in type-2 diabetes patients.

**Methods:**

A systematic review with meta-analysis. Computerised bibliographic database were searched up to January 2008 for randomised controlled trials evaluating group-based DSME for adult type-2 diabetics versus routine treatment where the intervention had at least one session and =/>6 months follow-up. At least two reviewers independently extracted data and assessed study quality.

**Results:**

In total 21 studies (26 publications, 2833 participants) were included. Of all the participants 4 out of 10 were male, baseline age was 60 years, BMI 31.6, HbA1c 8.23%, diabetes duration 8 years and 82% used medication. For the main clinical outcomes, HbA1c was significantly reduced at 6 months (0.44% points; P = 0.0006, 13 studies, 1883 participants), 12 months (0.46% points; P = 0.001, 11 studies, 1503 participants) and 2 years (0.87% points; P < 0.00001, 3 studies, 397 participants) and fasting blood glucose levels were also significantly reduced at 12 months (1.26 mmol/l; P < 0.00001, 5 studies, 690 participants) but not at 6 months. For the main lifestyle outcomes, diabetes knowledge was improved significantly at 6 months (SMD 0.83; P = 0.00001, 6 studies, 768 participants), 12 months (SMD 0.85; P < 0.00001, 5 studies, 955 participants) and 2 years (SMD 1.59; P = 0.03, 2 studies, 355 participants) and self-management skills also improved significantly at 6 months (SMD 0.55; P = 0.01, 4 studies, 534 participants). For the main psychosocial outcomes, there were significant improvement for empowerment/self-efficacy (SMD 0.28, P = 0.01, 2 studies, 326 participants) after 6 months. For quality of life no conclusion could be drawn due to high heterogeneity. For the secondary outcomes there were significant improvements in patient satisfaction and body weight at 12 months for the intervention group. There were no differences between the groups in mortality rate, body mass index, blood pressure and lipid profile.

**Conclusions:**

Group-based DSME in people with type 2 diabetes results in improvements in clinical, lifestyle and psychosocial outcomes.

## Background

Diabetes mellitus is one of the most common chronic disorders in the western world. In 2007, it was estimated that there were 246 million people (7.3% of adults aged 20-79) with diabetes compared to194 million in 2003 [1]. Type 2 diabetes constitutes about 85% to 95% of all diabetes cases in developed countries.

The World Health Organisation Report on therapeutic patient education recognises the importance of patient-centred education in the effective management of chronic diseases [2]. Therapeutic patient education is education designed to help a patient (or a group of patients and their families) to manage their treatment and prevent avoidable complications, while keeping or improving their quality of life. It has been recognised that adoption of self-management skills (i.e. the learned ability to perform and act competently) by persons with diabetes is necessary to enable them to manage their diabetes.

In diabetes self-management education (DSME), the close involvement of patients and care givers is encouraged. In contrast, traditional education is didactic in nature and tends to be delivered in lecture format. There are several initiatives to provide guidelines for DSME. The International Diabetes Federation has published 'International Curriculum for Diabetes Health Professional Education' International [3] and 'International Standards for Diabetes Education' [4]. In the United States of America a 'National Standards for Diabetes Self-Management Education' has also been developed and regularly updated [5]. In the UK, the Department of Health has published the “Structured Patient Education in Diabetes” [6].

However, there are considerable variations in the content and form of DSME and thus no standardised description can be given of the intervention. Educational programs are frequently defined as complex interventions where it is often difficult to define the 'active ingredient' framework [7]. If a program is shown to be effective, that may be due to any combination of the theoretical model used, the skills of the educator, the venue, the rapport between the participants and so on. However, if sufficiently homogeneous good quality complex interventions are systematically reviewed, the active ingredient is more likely to become apparent.

In a Diabetes UK commissioned review from 1998 on the educational and psychosocial interventions for adults with diabetes [8], six meta-analyses were identified [9-14], one review [15] and 57 published controlled trials. Thus, a large number of trials have been undertaken early, mainly in secondary care in the United States. More recent reviews have evaluated the effects of different types of self-management training in type 2 diabetes [16-27] Although there is evidence that self-management training is effective, most reviews called for further research by way of well-designed and long-term studies.

None of the above reviews have had a comprehensive evaluation of the effect of diabetes self-management education delivered in a group format. This was done for studies up to 2003 by Deakin et.al. [28] where it was found that group based DSME had a significant effect on clinical, lifestyle and psychosocial outcomes. Group based DSME is widespread due to being a cheaper method and the added advantages of having patient meet and discuss with each other.

The current review builds on the previous review [28]. The aim of this study was thus to assess effects of group-based DSME compared to routine treatment on clinical, lifestyle and psychosocial outcomes in type-2 diabetes patients.

## Methods

This was a systematic review with meta-analysis of randomised controlled trials (RCT's) comparing group-based diabetes self-management education with routine treatment, waiting list control or no intervention. Only studies that assessed outcome measures six months or more from baseline were included in this review.

### Participants

Studies which included adults diagnosed with type 2 diabetes. There were no criteria for how the type 2 diabetes should have been diagnosed, but ideally it should have been described. In order to be consistent with changes in classification and diagnostic criteria of the disease through the years, the diagnosis should have been established using the standard criteria that were valid at the beginning of the trial.

### Interventions

Studies were included if the intervention described was group-based education specific for people with type 2 diabetes and if the duration of education was a minimum of one session lasting for one hour. Furthermore, the control group must have been given the routine treatment (standard of care recommended), remained on a waiting list or received no intervention (i.e. the present healthcare was continued).

### Outcomes

The time points for measurement were divided into short term (6 months - range 4 to 8 months) and long term (12 months - range 9 to 16 months) and 2 years or more (range 17 months or more). The main outcomes were Clinical (metabolic control measured by glycated haemoglobin and fasting blood glucose), Lifestyle (diabetes knowledge and self-management skills) and Psychosocial (quality of life and empowerment/self-efficacy). The secondary outcomes were; Body weight; Body mass index (BMI); Blood pressure (systolic/diastolic); Lipid profile (total cholesterol, HDL cholesterol, LDL cholesterol, triglycerides); Patient treatment satisfaction and Death.

### Search and study selection

For studies that were published up 2003 we relied on the searches and assessments that were conducted for the existing Cochrane review on diabetes self-management education delivered in group format [28]. A new search of Ovid MEDLINE®, The Cochrane Library, EMBASE, ERIC and PsycINFO was done for publications from 2003 to week 2 in 2008. Resources were also hand-searched through reference lists of articles and other reviews and contact with experts in the field. An example of the search strategy used for electronic searches is given in appendix A.

Two reviewers independently scanned the titles, abstract sections and/or keywords of every record retrieved. Full articles were retrieved for further assessment if the available information suggested that the study met the inclusion criteria. If there was any doubt regarding the fulfilment of these criteria, the complete articles were retrieved. Any differences in opinion were discussed and, if necessary, resolved by a third party. Data extraction and data entry were performed independently in duplicate by two reviewers. Differences in data extraction were discussed and if necessary resolved by a third reviewer.

If data was missing in a published report, the reviewers tried to contact the first author. If the values given in the publications were not in a form that could be used in the meta-analysis, the values were recalculated if possible using the directions given in the Cochrane handbook [29]. If the standard deviations were not given for the follow up values, the baseline standard deviations were used. The re-calculations concerned converting fasting blood glucose and lipid level data from milligrams per decilitre (mg/dl) to mill moles per litre (mmol/l) and calculating the standard deviation for values where it was not provided.

### Analysis

The methodological quality of the trials was assessed independently by two reviewers using the Risk of Bias approach described in the Cochrane Handbook [30]. This involves a description and a judgement for random sequence generation, allocation concealment, blinding, incomplete outcome data, selective outcome reporting and other potential sources of bias. ‘Low risk’ indicating low risk of bias, ‘High risk’ indicating high risk of bias, and ‘Moderate risk’ indicating either lack of information or uncertainty over the potential for bias. Any disagreements about methodological quality were resolved by discussions.

To describe the weighted average participant at baseline for included studies, the mean baseline value for each study was multiplied with the number of participants in the study. This was summarised across the studies and divided by the total number of participants.

For all analyses the DerSimonian and Laird method provided in Review manager v5 was used. A random effect approach was chosen for all analyses because it was not likely that the underlying data represented one true effect due to the differences in the populations and interventions in the different studies. For analyses of continuous data where the same measurement was used across studies, the mean difference was calculated. If the same underlying concept was measured but different outcome measurements were used, standardised mean difference (SMD) was calculated. For analyses of categorical data odds ratio (OR) was calculated. At the outset, a meta-analysis was conducted for all relevant outcomes with more than one study reporting results. Mean outcome data at each time point were compared for the main analyses. Mean change from baseline to 12 months of HbA1c was used for the subgroup and sensitivity analyses.

To test for heterogeneity, I2, which describes the percentage of total variation across studies that was due to heterogeneity rather than sample error (chance) [31], was used. If I2 was from 60% and above, a sensitivity analysis was done by removing the studies contributing to the heterogeneity and reporting this result as well. In the representation of the analysis in the tables, all the studies are included.

Change in HbA1c from baseline to 12 months was used for the subgroup and sensitivity analyses. This was calculated using the difference in absolute value at 12 months and subtracting the baseline value. The standard deviation (SD) was calculated taking the average of the baseline and 12 months SD.

Separate analyses of the effect on HbA1c at 12 months were performed for the following subgroups:

1. Ethnicity. Studies with participants who were mostly non-Caucasian.

2. Used theoretical model. Studies that explicitly stated that they had a theoretical model underpinning the education programme.

3. Type of educators. Studies that had one type of educator that delivered the programme to the participants and for those that had more than one person engaged as an educator.

4. Primary care. Studies that were delivered in primary care.

5. Baseline HbA1c level. Studies where the mean HbA1c baseline value was 7% or more.

6. Follow-up. Studies where the intervention group received follow-up education sessions or telephone calls.

7. Length of delivery. Studies where delivery of education programme was completed in 5 months or less, between 6 and 10 months and in 10 months or more.

8. Total number of hours. Total hours of education provided excluding follow up sessions, divided in quartiles.

9. Attendance rate. Studies with overall attendance rate less than 70%.

10. Number of participants. Number of participants in each group session.

11. Family and friends. Studies including family and friends as participants.

12. Number of sessions. Total number of sessions provided excluding follow up sessions, divided in three groups.

Sensitivity analyses for each outcome were performed in order to explore the influence of the following factors on HbA1c at 12 months:

1. Language of publication English. Analysis of studies that were not translated (i.e. excluding studies which had been published in a foreign language and then translated to English).

2. Number of participants in the study. Analysis of studies with the total number of participants more than the median of all the studies.

3. Recalculated values. Analyses of studies where some of the values were recalculated because they were reported in a format not usable in a meta-analysis.

4. Risk of bias (Study quality). Separate analysis of studies which were scored as low, moderate or high risk of bias.

5. Drop out. Analysis of studies with less than 10% overall dropout rate.

## Results

**Figure 1:**
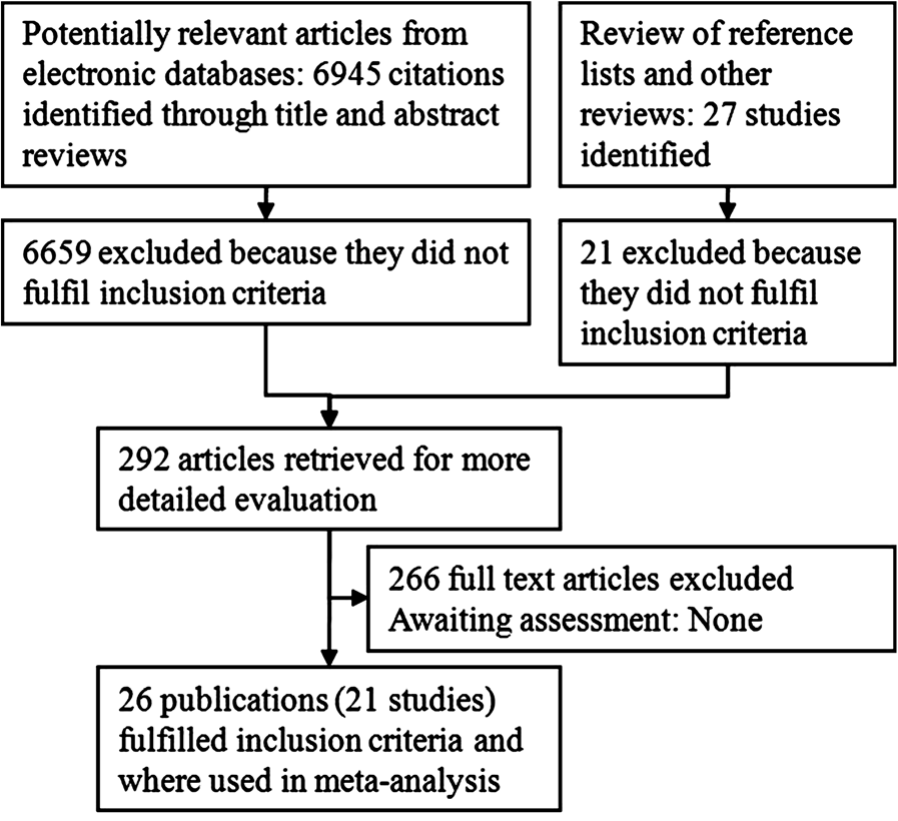
Stages of study identification

The original literature search conducted for the existing Cochrane review (to January 2003) [28] yielded 4598 citations and our updated search 2347 citations after excluding duplicates, giving a total of 6945 citations. A flow chart indicating the stages of study identification is included as Figure 1. A total of 292 citations either met the inclusion criteria or required sight of full paper before a decision could be made. Finally 266 publications were excluded and 21 studies reported in 26 publications were included.

Among the 266 publications that were excluded, five were included in the original Cochrane review (Domenech 1994; Domenech 1995; Kronsbein 1988; Pieber 1995; Rickheim 2002). They were excluded from the current review due to not being randomised trials. The reasons for the exclusion of the other publications included: lack of control group; length of follow-up being too short; absence of the pre-specified outcomes; intervention group in receipt of individual appointments in addition to the group-based education programme; delivery of group-based education programme to the control group; not all participants having type 2 diabetes; narrative papers, non-randomised trial, duplicate paper, irrelevant outcomes, results presented in format not usable in a meta-analysis, and group-based education programme that did not focus on diabetes self-management education. Several studies were excluded on more than one ground.

### Description of participations and studies

Twenty-one studies reported in 26 publications were included [32-56] (Table 1). Among these, five reported on the same studies by either reporting other outcome measures [50] or long term follow-up data [52-54]. In the first review; data from one unpublished study were included [57]. For this review, this has been replaced with the published data of the same study [38]. One publication reporting on a 3 armed study had data for the intervention groups combined to create one intervention group [44].

**Table 1:** Characteristics of studies included in the systematic review of randomized controlled trials assessing group based diabetes self-management education compared to routine treatment for people with type 2 diabetes mellitus

**Author Year, Country,**	**Number of participants recruited / at follow-up**	**Mean age (SD or range) years**	**Mean duration of diabetes (years)**	**Mean (SD) % HbA1c at baseline (Intervention / Control)**	**Inclusion criteria**	**Intervention**	**Follow-up (months or years)**	**Risk of bias**
Adolfsson 2007, Sweden	101/88	63.0 (9.0)	6.6 (4.1)	7.1 (1.0)/ 7.1 (0.8)	Diabetes type 2 duration of at least 1 year, receiving dietary or oral anti-diabetes treatment	4-5 sessions of 2.5 hours each and one follow-up session within 28 weeks, (12.5 hours), by trained physicians and diabetes specialist nurses	1 year	Low
Baradaran 2006, UK	118/80	58.5 (12.0)	9.0 (7.5)	Not stated	South Asians, type 2 diabetes, > 30 years of age	3 sessions of 2,5 hours + 25 minutes information video (7.5 hours), by dietician and podiatrist	6 months	Moderate
Brown 2002, USA					Mexican Americans	52 hours over 12 months (3 mths of weekly 2 hrs sessions, 6 mths of biweekly 2 hrs sessions, 3 mths of monthly 2 hrs sessions), by nurse, dietician & community worker	1 year	High
					35-70 years, diagnosed with Type 2 diabetes, 2 measures of FBG > 140 mg/dl, taken insulin or oral hypoglycaemic agents for >1 year			
Cabrera-Pivaral 2004, Mexico,	49/49	58.0 (10.6)	10.0 (10.4)	Not stated	Hispanics, obese type 2 diabetics	82 hours over 9 months (2 hours/week), by social worker and nutritionist	9 months	High
Clancy 2007, USA	186/159	56 (8.9)	Not stated	9.3 (2.0)/ 8.9 (2.1)	African Americans with poorly controlled diabetes type 2 (HbA1c > 8.0 %),	1 group visit/month for 12 months, 2 hours/visit (24 hours), by physician and registered nurse.	1 year	Moderate
Cooper 2003, UK	89/Not stated	58 (30-73)	6 (1-30)	7.9 (1.7)/ 7.0 (1.6)	Type 2 diabetics, oral hypoglycaemic agents treatment	8 weekly sessions for 2 hours (16 hours), by diabetes specialist nurses	1 year	Moderate
Deakin 2006, UK	314/291	61.6 (10.4)	6.7 (6.6)	7.7 (1.6)/ 7.7 (1.6)	Adults with type 2 diabetes identified from practice registers using WHO criteria	2 hours per week for 6 weeks (12 hours), by diabetes research dietician/diabetes educator	14 months	Low
Heller 1988, UK	87/75	56.4 (8.5)	Not stated	12.3 (2.8)/ 12.7 (2.5)	Newly diagnosed age 30-75 yrs	4.5 hr for 3 consecutive weeks, 1.5 hr at both 3 & 6 months (7.5 hours), by diabetes nurses and a dietician	1 year	Moderate
Holtrop 2002, USA	132/88	61.5 (No SD or range)	Not stated	8.0 (1,34)/ 7.7 (1,34)	More than 40 years, female type 2 diabetics, HbA1c > 7 % in past 6 months	Six weekly 1 1/2 hour sessions (9 hours), by trained lay health advisors	6 months	Moderate
Hornsten 2004, Sweden	104/99	63.5 (9.2)	Not stated	5.7 (0.8)/ 5.8 (0.7)	Aged 40 - 80 years, diagnosed with type 2 diabetes during the previous 2 years. Oral, guts and/or insulin, and diet treatment	10 group sessions, 2 hours/session for 9 months (20 hours), by diabetes nurses	1 year	Moderate
Lozano 1999, Spain	243/234	64.3 (11.2)	8.6 (8.3)	6.6 (1.4)/ 6.7 (1.3)	Type 2 diabetes	1 hr 30 min/day for two consecutive days (3 hours), and further education in year two, by nurses	2 years	Moderate
Lujan 2007, USA	149/140	Not stated	Not stated	8.2 (2.2)/ 7.7 (1.5)	40 years of age or older, Self reported Mexican-American ethnicity, Diagnosed with type 2 diabetes for at least 1 year, Taking or having taken hypoglycemic agents within the past 6 months.	2 hour long sessions for 8 weeks (16 hours) plus biweekly telephone, by Promotoras (Community lay workers)	6 months	High
Mayer-Davies 2004, USA	152/ Not stated				Clinical diagnosis of diabetes, BMI of 25 kg/m2 or greater during the previous calendar year	Intense: 1 hour weekly for four mths, biweekly for two mths and once every moth for six mths, by Nutritionist	1 year	High
						Reimbursable: Four 1 hour sessions (3 group sessions & 1 individual session) over 12 months, by Nutritionist		
McKibbin 2006, USA	64/57	54.0 (9.3)	8.7 (6.2)	7.4 (2.9)/ 6.7 (2.1)	Older schizophrenic patients. Diet, Oral agents, and Insulin treatment	24 weekly 90 minute sessions for 6 months (36 hours).	6 months	Moderate
Pennings-van der 1991, Netherlands	118/83	64.4 (9.6)	10.9 (7.6)	8.0 (1.8)/ 7.6 (1.5)	Diabetes mellitus type Il treated with diet and/or oral agents	7 half-day meetings in 7 weeks, by physicians, dieticians, diabetologist, diabetes nurse	8 months	High
Rosal 2005, USA	25/Not stated	62.6 (8.7)	8.2 (5.6)	7.7 (1.2)/ 9.3 (1.8)	Hispanics. Diet, Oral hypoglycemic agents, Insulin, Alternative medicines treatment	Initial 1 hour individual session, followed by 10 weekly 2.5 to 3 hour group sessions and two 15 minute individual sessions that occurred immediately prior to the group session (30 hours), by Diabetes nurse, Nutritionist and an Assistant	6 months	High
Sarkadi 2004, Sweden	77/64	66.4 (9.3)	4.3 (4.0)	6.5 (1.5)/ 6.4 (1.5)	Type 2 diabetes, treated with insulin only for 2 year or less	Meetings every months over 12 months, by Pharmacists assisted by Diabetes nurse specialists the two first meetings	2 years	Moderate
Toobert 2003/2007, USA	279/215	60.9 (7.9)	8.3 (7.7)	7.4 (1.3)/ 7.4 (1.5)	Female, Diagnosis of type 2 diabetes for at least 6 months, Postmenopausal,	3 days non-residential retreat, followed by 4-hour weekly meetings for 6 months	6 months [49]; 24 months ([50])	Moderate
Trento 1998/2001/2002/2004, Italy	112/84	61.5 (30-80)	9.6 (1-39)	7.2 (1.3)/ 7.3 (1.4)	Treated with diet or oral hypoglycaemic agents, followed in clinic >1 year	Every 3 months for 1 year (4 x 60/70 min), and 15-30 min. individually for those who developed specific problems[51]; every 3 months for 2 years (1 hour x 8 = 8 hr/2 yr) ([52]); every 3 months for 2 years and 7 sessions in year 3 +4 (total 15 hrs/4 yrs) ([53]); every 3 months for 2 years and 7 sessions in year 3and 4 (total 15 hrs/4 yrs) and started again in year 5 ([54]), by Two physicians and educationist	1 year [51], 2 years ([52]), 4 years ([53]), 5 years ([54])	Moderate
Wattana 2007, Thailand	157/147	56.8 (10.1)	6.2 (5.0)	8.1 (1.9)/ 8.1 (2.0)	35 years or older, Diagnosed with type 2 diabetes for at least 6 months, Fasting plasma glucose level >140 mg for at least 2 follow up visits, Asian participants. Oral hypoglycaemic agents treatment	120 minutes small group diabetes education class, 4 small group discussions (90 mins/group), two 45 minutes home visits by the researcher	6 months	Moderate
Zapotoczky 2001, Austria	36/36	57.5 (9.8)	Not stated	8.6 (1.6)/ 8.0 (1.5)	Not stated	1.5 hours monthly for 10 months (15 hours) delivered by a dietician	1 years	High

A total of 2833 participants were included in the 21 studies with 1454 (51%) in the intervention group. Four out of ten participants were male and at baseline the pooled average age was 60 years (SD 9.5), BMI 31.5 (SD 5.6), diabetes duration was 8.1 years (SD 7.0), HbA1c level 8.23% (SD 1.80), and 81.9% used insulin and/or oral glucose lowering agents. The analyses showed that there were no significant differences between the intervention and control group at baseline (Table 2). The pooled average HbA1c level in the intervention group was 8.31 (SD 1.83) and in the control group 8.16 (SD 1.76); a non-significant weighted mean difference of 0.09 (95%CI -0.05 to 0.23, p = 0.21) (Table 2, Analysis 1.5).

**Table 2:** Baseline values

**Analysis number / Outcome**	**Effect measure**	**N studies**	**N participants (Int/contr)**	**Diff (95%CI)**	**P-value**
1.1 Sex (% males)	Odds Ratio	17	1029/983	0.92 (0.76 to 1.10)	0.355
1.2 Age	Mean Diff	17	1204/1128	-0.02 (-0.96 to 0.92)	0.967
1.3 Body mass index	Mean Diff	16	1152/1081	0.09 (-0.39 to 0.57)	0.712
1.4 Duration of disease (years)	Mean Diff	13	1006/913	-0.18 (-0.98 to 0.63)	0.670
1.5 HbA1c	Mean Diff	19	1370/1276	0.09 (-0.05 to 0.23)	0.213
1.6 Use of OHA and/or Insulin	Odds Ratio	12	748/691	0.91 (0.68 to 1.23)	0.553

Table 1 contains the characteristics of all the included studies.

Three of the 26 publications required translation; two were written in Spanish [35,42] and one in Dutch [46]. Eight studies were carried out in the United States [34,36,40,43-45,47,49,50], four in the United Kingdom [33,37-39], three in Sweden [32,41,48], one in Austria [56], one in Mexico [35], one in Thailand [55], one in Spain [42], one in the Netherlands [46] and one in Italy [51-54].

The length of follow-up was six months for 8 of the trials [33,40,43,45-47,49,55], 12 months for 11 of the trials [32,34-39,41,44,51,56], and two years for two trials [42,48]. The Toobert study had follow-up at two years [50] and the Trento study also had follow-up at two years [52], four years [53] and five years [54]. The duration of the DSME varied with the least intensive being three hours per year for two years [42]. Ten trials described programmes that ranged from six to twenty hours of group-based education over a period of between four weeks and 10 months [32,36-41,43,46,56]. The most intensive education programmes were 52 hours over one year [34], 36 hours over 6 months [45], 30 hours over 2.5 months [47] and 96 hours over 6 months [50].

The settings in twelve of the 21 studies were primary care [32-34,36,38,41,42,44-47,49,50], and five were delivered in hospital diabetes centres [39,43,51-56]. Four studies did not indicate the settings in which the education programmes were held [39,43,51-56].

The educators were all health professionals, with the exception of two studies where the educators were lay health advisors or community workers [40,43]. Four of the DSMEs were delivered by physicians in association with other health personnel [32,36,46,51-54]. Four DSMEs were delivered by a dietician and a nurse [33,34,39,49,50] with some also involving community workers and other health personnel [33,34,49,50]. Two programmes were delivered by dieticians working alone [38,56], one by a nurse working alone [42] and one by a nutritionist working alone [44]. Four studies reported the programmes being delivered by diabetes specialist nurses in collaboration with other health personnel [37,41,47,48].

Four studies reported that a family member or friend also was invited to attend the programme [34,39,46,51-53].

The theoretical model used to plan the DSME was reported in ten studies. Five studies described single theories while the remaining five used a combination of theories. The single theories were social cognitive theory [45,47], empowerment model [32,43], and systematic education approach [51-54]. The combination of theoretical models were the empowerment model, theories of planned behaviour and personal models of sickness [37], the empowerment model and the discovery learning theory [38], the social cognitive theory and the social ecological theory [50], the self-efficacy and self-management theories [55] and the cognitive theory and operant reinforcement theory [56]. One study stated that the DSME was 'participatory' [42].

The number of participants in each group was reported in ten studies [32,33,36,38,39,43,46,50-54,56]. The smallest group comprised five to eight participants [32] and the largest group comprised 40 patients per session [50].

In one study [56], both the intervention and the control groups attended a 4-week education programme before randomisation. The intervention group was then given additional 15 hours of education (1.5 hour monthly for 10 months).

The control groups were placed on waiting lists to receive the DSME after the study in four studies [34,37,48,55]. In sixteen studies, the comparison group received routine treatment [32,33,36,38-47,49-54,56]. It was not clear what type of routine treatment was offered to the control group participants in one study [35]. Routine treatment was defined differently among the studies; as twice-a-year appointments with the physicians and diabetes specialist nurses for biomedical tests and examinations in accordance with regional diabetes guidelines based on the Swedish National Guidelines [32], separate one-hour-long individual appointments with a dietician, practice nurse and general practitioner [38], one or two visits per year with the respective diabetes nurses [41] and one-on-one patient education by clinical staff during scheduled medical follow up visits, which consisted of verbal information and one or two pamphlets on self-management skills [43]. It also involved one individual session by a study nutritionist at the beginning of the study [44], usual care with three brochures from American Diabetes Association [45], 15 to 20 minutes with a multidisciplinary diabetes team every three months approach [51-54] or an individual appointment with a dietician every three months [56].

Twelve publications had some sections of their results recalculated before input into the meta-analysis [34-37,39,40,42,44,45,48,49,54]. Two studies [34,35] reported their fasting blood glucose results in mg/dl. For inclusion in the meta-analysis, these were converted to mmol/l. One study [36] did not report the standard deviations with the findings while another [37] did not report the standard deviation for the treatment satisfaction results. One study [39] reported the results as means with the standard error of the mean and another [40] presented some outcomes without standard deviations and reported p-values without presenting the actual data. One study [48] presented results in form of graphs with means and confidence intervals and another [42] reported some outcomes with means and confidence intervals and others with means and standard deviations. One study [47] reported the findings as mean changes from baseline with standard deviations and another [44] also reported the findings as mean changes from baseline but did not give standard deviations. Five studies [44,45,47,49,54] reported their lipid profile findings in mg/dl and these were converted to mmol/l for analysis.

All trials included in the review except two [33,35], assessed glycated haemoglobin (HbA1c) at some time point.

The studies that recorded the number of deaths did not identify whether or not the deaths were diabetes related. Only one study recorded diabetes complications (creatinine, albuminuria, diabetic retinopathy, foot ulcers) at two years [52] and four years [53].

General quality of life was assessed using Average quality of life questionnaire [38,58]; SF 12 Mental and physical health [49] and SF 36 Thai version [55]. Disease specific quality of life was measured using the Audit of diabetes dependent quality of life [38,47]; Diabetes Distress Scale [50]; Diabetes Quality of Life [54]; Problem Areas in Diabetes (PAID) Social and Self-care [49] and Diabetes Symptom Checklist (DSC-R) [41].

Diabetes knowledge was assessed using General knowledge of diabetes questionnaire [43,46]; Confidence in diabetes knowledge scale [32]; Knowledge, Attitudes and Practices scores [33]; Lifestyle measure [38]; Diabetes knowledge [45]; Audit of diabetes knowledge scale [47] and Knowledge of diabetes questionnaire [54].

Diabetes self-management skills were assessed using Self-care activities and food frequency questionnaire [37,38,46]; Health behaviour conduct and Problem solving ability [51-54]; Self-monitoring of blood glucose levels [42,47] and Stages of change questionnaire [40].

Self-efficacy/empowerment was assessed using Self efficacy-plate model [32]; Diabetes empowerment scale [38,45]; Self efficacy scores for diet, exercise, self-monitoring, oral glycaemic agents, insulin [47]; Confidence to overcome challenges [50] and Sallies self-efficacy for diet and exercise [50].

Satisfaction with treatment was assessed using Satisfaction with daily life [32]; Patient treatment satisfaction [36]; Diabetes treatment satisfaction questionnaire [38,41] and Personal models of disease questionnaire [37].

A cost-effectiveness analysis was performed at a four year follow-up for one study [53] and the cost of delivering the programme was estimated in two studies [34,50].

Ten studies presented a power calculation and based recruitment numbers on the calculation [32,36-38,42-44,48,50,55] and all of these managed to reach their target sample sizes.

### Risk of bias

Based on the quality criteria described in the methods section, two studies were classified as having a low risk of bias [32,38], 12 studies as having moderate risk of bias [33,36,37,39-42,45,48-55], and seven studies were classified as having a high risk of bias [34,35,43,44,46,47,56].

The methods of sequence generation were elaborated in five studies [33,36,38,48,51-54]. One study stated that the sequence generation was conducted using permuted random blocks [38], while another used identical envelopes containing participants details put in a box and randomly assigned to intervention or control group [48]. The Trento study used random number tables for sequence generation [51-54]. One study stated that minimization was performed [33] and another stated that the sequence generation was computerised [36]. Eleven studies did not provide the details of how sequence generation was conducted, although they made reference to it by stating that participants were randomly allocated to either intervention or control groups [34,35,37,39,40,42,44-47,56]. In five studies [32,41,43,49,50,55], the methods of sequence generation were not mentioned.

Allocation concealment was done using sealed opaque envelopes in three studies [32,38,48]. In one study [41], randomisation was done at clinic level, to allocate clinics to the intervention group and to the control group, implying that most probably there was no allocation concealment. In another study [37], it was mentioned that participants were blindly and randomly allocated, implying that most probably allocation concealment was done. The rest of the studies did not refer to allocation concealment in their reports [33-36,39,40,42-47,49,50,55,56].

Only one study made reference to attempts made towards blinding of treatment [38]. The study attempted to blind the control group to the fact that they were the controls by presenting 'routine treatment' as an individual appointment intervention. Trento [52,53] reported that the physicians who were in charge of group sessions also attended to the control participants during the general diabetes clinics, but were blinded to the control participants’ status in the study to avoid performance bias. Only one study [38] reported that outcome assessment was performed by a community nurse and health care assistant, who were both blinded to treatment assignment.

Thirteen studies were considered not to have any incomplete outcome data reported. Ten of these ensured that data of participants that dropped-out from the studies before the end were reflected in the analysis [32,36,39,41-43,45,46,48,55]. Eight stated that their analyses were by intention to treat [38,40,49-54]. Two studies had some incomplete outcome data [33,34]. One of the studies [33] stated that the study lost 15 (25 %) out of 59 intervention participants and 23 (39 %) out of 59 control participants at 6 months follow up. However, intention to treat analysis was not stated and the data for the participants that dropped-out was not included in the outcome tables. The other study [34] also did not mention that intention to treat analysis was conducted and did not show the numbers of participants included in the analysis to give an indication whether the ones who dropped-out were included in the analysis or not. For the remaining six studies, it was unclear whether the issue of incomplete outcome data was sorted out or not. Five studies [35,37,44,47,56] did not indicate whether their studies had lost some participants to follow-up and neither did they mention that intention to treat analyses were conducted.

All the studies measured and reported all the pre-specified outcomes except for two studies [36,44]. One of the studies [36] did not report on glycated haemoglobin at six months, and blood pressure and lipid profiles at six and 12 months, and the other [44] only reported weight change at 12 months follow-up, and did not report on the other outcomes at 12 months follow up (HbA1c, BMI, Cholesterol level, Blood pressure).

Quality assessment of most of the studies led to the conclusion that there was possibility for bias due to missing information and unclear issues. However, for five studies [32,38,41,42,48], there were no further issues of concern identified that could be deemed possible causes of bias.

### Effects – main outcomes

Thirteen studies [34,37-40,43-49,55] involving 1827 participants assessed glycated haemoglobin (HbA1c) at six months. The mean difference was -0.44 percentage points (95% CI: -0.69 to -0.19, P = 0.0006, Table 3: Analysis 3.1) in favour of DSME with a heterogeneity of I2 = 56%. At the 12 months' follow-up, the meta-analysis of 11 studies [32,34,36-39,41,42,48,51,56] including 1503 participants gave a mean absolute difference of -0.46 percentage points (95% CI: -0.74 to -0.18, P = 0.001, Table 3: Analysis 3.2) with a heterogeneity of I2 = 65%. The heterogeneity was due to two outlying studies [37,41] which reported the highest reduction in HbA1c in favour of the DSME of 1.0 percentage points [41] and the highest increase of 0.7 percentage points [37]. Removing these, the nine remaining studies showed an overall significant reduction in HbA1c of -0.50 percentage points (95% CI: -0.73 to -0.27, P < 0.0001, I2 = 33%). Using changes in HbA1c from baseline to twelve months for all the 11 studies, gave a change in HbA1c in favour of DSME of -0.55 percentage points (95% CI: -0.75 to -0.35, P < 0.00001, I2 = 37%), thus reflecting the slight non-significant difference in baseline values between the groups (Table 2: Analysis 1.5). Three studies involving 397 patients assessed glycated haemoglobin at two years [42,48,52] with no heterogeneity between the studies (I2 = 0%). There was a significant reduction in HbA1c for the patients allocated to the DSME compared to the control group (-0.87 percentage points; 95% CI: -1.25 to -0.49, P < 0.00001, Table 3: Analysis 3.3).The Trento study also assessed glycated haemoglobin at four [53] and five [54] years' follow-up. The four year follow-up involved 90 patients and found a significant reduction in the group education group compared to the control group (-1.6 percentage points; 95% CI: -2.3 to 0.9, P < 0.00001). The five year follow-up involved 112 participants and reported reduction of 1.7 percentage points in the intervention group compared to the control group (95% CI: -2.19 to -1.21, P = 0.00001).

**Table 3:** **Meta-analysis of primary and secondary outcomes of group-based diabetes self-management education programme with comparison for intervention (Int) and control (contr) groups and the heterogeneity (measured by I**^**2**^**) of the analyses**

**Analysis number / Outcome**	**Effect measure**	**N studies**	**N participants (Int/contr)**	**Difference (95%CI)**	**P-value**^**A**^	**Hetero-geneity (I2)**
3.1 Glycated haemoglobin (6 months)	Mean Diff	13	977/850	-0.44 (-0.69 to -0.19)	0.001	55.8
3.2 Glycated haemoglobin (12 months)	Mean Diff	11	750/753	-0.46 (-0.74 to -0.18)	0.001	64.6
3.3 Glycated haemoglobin (2 years)	Mean Diff	3	199/198	-0.87 (-1.25 to -0.49)	0.000	0.0
3.4 Fasting blood glucose (6 months)	Mean Diff	3	206/195	-0.73 (-2.22 to 0.76)	0.336	68.1
3.5 Fasting blood glucose (12 months)	Mean Diff	5	344/346	-1.26 (-1.69 to -0.83)	0.000	0.0
3.6 Diabetes knowledge (6 months)	Std Mean Diff	6	390/378	0.69 (0.43 to 0.96)	0.000	63.5
3.7 Diabetes knowledge (12 months)	Std Mean Diff	5	477/478	0.85 (0.48 to 1.22)	0.000	85.5
3.8 Self management skills (6 months)	Std Mean Diff	4	295/239	0.55 (0.11 to 0.99)	0.015	79.1
3.9 Quality of life (6 months)	Std Mean Diff	3	242/231	0.31 (-0.15 to 0.78)	0.186	77.1
3.10 Self efficacy/Empowerment (6 months)	Std Mean Diff	2	167/159	0.28 (0.06 to 0.50)	0.012	0.0
3.11 Weight (6 months)	Mean Diff	3	216/217	-2.08 (-5.55 to 1.39)	0.239	48.2
3.12 Body Mass Index (6 months)	Mean Diff	7	633/526	-0.21 (-0.86 to 0.43)	0.514	0.0
3.13 Weight (12 months)	Mean Diff	4	247/245	-1.66 (-3.07 to -0.25)	0.021	0.0
3.14 Body Mass Index (12 months)	Mean Diff	7	538/554	-0.22 (-1.13 to 0.69)	0.634	62.2
3.15 Systolic blood pressure (6 months)	Mean Diff	5	454/360	-0.34 (-5.19 to 4.51)	0.891	67.9
3.16 Diastolic blood pressure (6 months)	Mean Diff	5	454/360	-0.46 (-2.31 to 1.39)	0.627	26.6
3.17 Systolic blood pressure (12 months)	Mean Diff	2	168/159	-2.61 (-6.74 to 1.52)	0.216	0.0
3.18 Total cholesterol (6 months)	Mean Diff	7	632/529	-0.04 (-0.17 to 0.10)	0.605	0.0
3.19 Triglycerides (6 months)	Mean Diff	7	632/529	-0.16 (-0.35 to 0.03)	0.104	0.0
3.20 Total cholesterol (12 months)	Mean Diff	4	324/332	0.07 (-0.09 to 0.24)	0.377	0.0
3.21 Triglycerides (12 months)	Mean Diff	4	325/332	0.03 (-0.42 to 0.48)	0.883	79.7
3.22 High density lipoprotein (6 months)	Mean Diff	6	515/417	0.02 (-0.05 to 0.08)	0.623	0.0
3.23 Low density lipoprotein (6 months)	Mean Diff	6	515/417	-0.05 (-0.20 to 0.10)	0.528	0.0
3.24 Treatment satisfaction (6 months)	Std Mean Diff	2	205/185	0.65 (0.44 to 0.85)	0.000	0.0
3.25 Treatment satisfaction (12 months)	Std Mean Diff	3	247/237	0.39 (0.21 to 0.57)	0.000	0.0
3.26 Death	Odds Ratio	4	351/349	1.10 (0.37 to 3.29)	0.867	3.2

A. The P- value is calculated for the difference between the intervention and control group.

Three studies [34,45,46] with a total of 401 participants reported on fasting blood glucose levels at six months. With all the studies included, the heterogeneity was I2 = 68% and the mean difference was -0.73 mmol/l (95% CI: -2.22 to 0.76, P = 0.34, Table 3: Analysis 3.4). However, removing one study [46] reduced the heterogeneity to I2 = 0% and a mean difference in favour of the DSME of -1.53 mmol/l (95% CI: -2.37 to -0.69, P = 0.0004) was observed. The baseline difference between the groups in the removed study [46] were 0.42 and although this increased to 0.66 at eight months, the difference between this study and the other two became larger (and thereby increasing heterogeneity) due to the difference in baseline value. Thus, due to the problem of heterogeneity, no conclusion can readily be drawn about the result of fasting blood glucose at 6 months. At 12 months, five studies assessed fasting blood glucose [34,35,39,42,51] with no heterogeneity between studies (I2 = 0%). There was an overall significant improvement in patients allocated to the DSME compared with those in the control group (-1.26 mmol/l; 95% CI: -1.69 to -0.83, P < 0.00001, Table 3: Analysis 3.5). Two studies assessed fasting blood glucose at two years [42,52]. The larger of the two studies [42] involving 243 participants showed a significant improvement of fasting blood glucose in favour of the DSME (difference 1.8 mmol/l; 95% CI: 1.2 to 2.4, P < 0.00001) but the other study [52] involving 80 participants did not show a significant improvement in the DSME over the control group (difference 0.7 mmol/l; 95% CI: -0.4 to 1.9, P = 0.24). However, the Trento study reported a significant difference between groups at the four years' follow-up [53] in favour of the DSME (difference 1.7 mmol/L; 95% CI: 0.2 to 3.2, P = 0.03), while the difference between the groups at the five year follow-up [54] was not significant (-0.10%, 95% CI: -1.18 to 0.98, P = 0.86).

Six studies with a combined total of 768 participants measured diabetes knowledge at six months [33,38,43,45-47]. As the studies had used different validated questionnaires to measure knowledge, the standardised mean difference method of analysis was used. Heterogeneity between the studies was I2 = 64% (SMD 0.69; 95% CI: 0.43 to 0.96, P < 0.00001, Table 3: Analysis 3.6). When one study [33], which reported the least increase in knowledge among the intervention group was removed, the heterogeneity was removed (I2 = 0 %) giving a SMD of 0.83 (95% CI: 0.67 to 0.99, P < 0.00001). At 12 months five studies involving 955 participants measured diabetes knowledge [34,38,39,42,51]. However, there was significant heterogeneity (effect size/SMD 0.85; 95% CI: 0.48, 1.22, P < 0.00001, I2 = 85%, Table 3: Analysis 3.7). Three studies had to be removed to reduce the heterogeneity [34,38,39], two large ones with lower SMD and one smaller with higher SMD. The meta-analysis with the remaining two studies [42,51] with a combined total of 333 participants gave a SMD of 1.03 (95% CI: 0.8 to 1.26, P < 0.00001, I2 = 0%). Two studies measured diabetes knowledge at two years. There was, significant heterogeneity (I2 = 97%) and a meta-analysis was not performed. Both studies showed significant better knowledge for the intervention group [42]: SMD 2.31; 95% CI: 1.99 to 2.64, P < 0.00001; [52]: SMD 0.86; 95% CI: 0.47 to 1.24, P = 0.0001). At four and five years follow up, Trento [53,54] measured diabetes knowledge and found that increased diabetes knowledge remained in the patients allocated to the DSME [53]: SMD 1.27; 95% CI:0.82 to 1.73, P < 0.00001; [54]: SMD 1.36; 95% CI: 0.95 to 1.77, P = 0.00001).

Seven studies measured some aspect of self-management [38,40,42,46,47,49,51-54]. However, only four studies involving 534 participants had data that could be used in a meta-analysis using standardised mean difference at six months [38,46,47,49]: SMD 0.55 (95% CI: 0.11 to 0.99, P = 0.01, I2 = 79%, Table 3: Analysis 3.8. Removing one study [47], which had the highest effect size, eliminated the heterogeneity (I2 = 0%) and resulted in a SMD of 0.29 (95% CI: 0.11 to 0.46, P = 0.002). One study [38] reported that at 14 months, self-management scores had remained significant in respect of exercise (P = 0.02) and foot care (P = 0.003) but there was no significant difference between the groups for self-monitoring of blood glucose levels (P = 0.17). For food intake, there were trends suggesting that the participants invited to the group intervention compared to those in the control group were consuming more percentage energy from carbohydrate (difference 3.3%; 95% CI: 0.3 to 6.9, P = 0.07), more energy from total sugars (difference 6.6%; 95% CI: 3.4 to 9.9, P < 0.001), less energy from total fat (difference 2.7%; 95% CI: 0.3 to 5.6, P = 0.08), less energy from saturated fat (difference 1.1%; 95% CI: 0.0 to 2.3, P = 0.05) and an extra 2 portions of fruit and vegetables per day (difference 2.2 portions; 95% CI: 1.1 portions to 3.2 portions, P < 0.001). The other three studies not included in the analysis provided narrative reports of their findings on self-management. One study [42] measured the percentage of participants who carried out self-monitoring of blood glucose levels and found a significant difference between the two groups in favour of the DSME at both one and two years (P < 0.005). The second study [40] reported that the group programme participants made positive improvement in stages of change for five behaviours: physical activity (P = 0.003); reduction of high fat foods (P = 0.008); consumption of five portions of fruit and vegetables(P < 0.0001); consumption of three meals daily (P = 0.09); limitation of refined sugar intake to one product per day or less (P = 0.001). However, the statistical analysis was performed on pre-test means versus post-test means for the intervention group and no data was provided for the control group. Trento developed and validated a health behaviours questionnaire and reported that the score was significantly greater for the group education participants than for the controls at one year ([51], P < 0.005), two years ([52], P < 0.001), four years ([53], P < 0.001) and five years ([54], P < 0.00001).

Three studies with 473 participants measured quality of life at six months [38,47,55]) using different questionnaires. Heterogeneity between the studies was high with no differences between the groups (SMD 0.31; 95 % CI: -0.15 to 0.78, P = 0.19, I2 = 77%, Table 3: Analysis 3.9). The heterogeneity was due to one study [38] and removing this study removed heterogeneity and gave a significant SMD of 0.57 (95% CI: 0.27 to 0.88, P = 0.0003, 172 participants). The removed study [38] found no overall improvement in overall quality of life but in the sub-scales there were significant improvement for the DSME: freedom to eat (difference 1.7; 95% CI: 0.8 to 2.5, P < 0.001); enjoyment of food (difference 1.2; 95% CI: 0.2 to 2.1, P = 0.046); and freedom to drink (difference 1.5; 95% CI: 0.4 to 2.5, P = 0.005). Thus, due to the problem of heterogeneity, no conclusion can readily be drawn about the result of overall quality of life at 6 months. At 12 months two studies measured quality of life [38,51]. The first study [38] reported similar results to those at four months, namely no significant improvement in overall quality of life, but significant improvements for the sub-scales: freedom to eat (difference 1.1; 95% CI: 0.2 to 2.1, P = 0.04); enjoyment of food (difference 1.1; 95% CI: 0.1 to 2.0, P = 0.05); and freedom to drink (difference 1.5; 95% CI: 0.5 to 2.6, P = 0.01). The second study [51] did not find a significant difference in quality of life at 12 months but reported a significant improvement in quality of life at two years ([52], P < 0.001), at four years ([53], P < 0.009) and at five years ([54], P < 0.00001).

Two studies [38,47], with a total of 326 participants assessed the level of empowerment and psychosocial self-efficacy at six months. As the studies had used different questionnaires the standardised mean difference (SMD) was used. There was no heterogeneity between the studies (I2 = 0%), and improvement in self-efficacy among the intervention groups was 0.28 above that of the control groups (95% CI: 0.06 to 0.50, P = 0.01, Table 3: Analysis 3.10). Only [38] assessed empowerment scores at 12 months, and the scores were still significantly higher amongst patients allocated to the DSME: the total empowerment (difference 0.3; 95% CI: 0.04 to 0.6, P = 0.006); psychosocial adjustment to diabetes (difference 0.3; 95% CI: 0.02 to 0.7, P = 0.005); readiness to change (difference 0.3; 95% CI: 0.1 to 0.5, P = 0.001); and setting and achieving goals (difference 0.2; 95% CI: 0.05 to 0.4, P = 0.02).

### Effects – secondary outcomes

Three studies, having a combined total of 433 participants, assessed body weight at six months [38,39,45]. The heterogeneity for the three studies was I2 = 48%. Overall reduction in body weight was 2.08 kg more than in the control group but the difference was not statistically significant (95% CI: -5.55 to 1.39, P = 0.24, Table 3: Analysis 3.11). Seven studies involving 1159 participants assessed BMI at six months [34,38,44-47,49] with no heterogeneity between studies (I2 = 0%). There was a difference between groups of 0.21 kg/m2 in favour of group education but, as in the case of body weight, that difference was not statistically significant (95% CI: -0.86 to 0.43, P = 0.51, Table 3: Analysis 3.12). At 12 months four studies, involving 492 patients, assessed body weight [38,39,51,56] with no heterogeneity between studies (I2 = 0%). The mean difference between the DSME and control group was 1.66 kg (95% CI: -3.07 to -0.25, P = 0.02, Table 3: Analysis 3.13). Also at 12 months, seven studies with a total of 1092 participants assessed BMI [32,34,35,38,41,42,51] with a heterogeneity of I2 = 62%. There were no significant difference between the groups (-0.22 kg/m2; 95% CI: -1.13 to 0.69, P = 0.63, Table 3: Analysis 3.14). Heterogeneity was caused by one study [35] but removing this did not change the results (0.12 kg/m2 (-0.67, 0.91), P = 0.76, I2 = 35%). One study [38] measured waist circumference at both four and 14 months. There was no significant difference between the two groups at four months (difference 1.3 cm; 95% CI: -1.8 to 4.1, P = 0.44) but there was a trend in favour of the DSME at 14 months (difference 2.8 cm; 95% CI: -0.3 to 5.6, P = 0.06).

Five studies including 814 participants measured systolic and diastolic blood pressure at six months [38,44,45,47,49] Heterogeneity between the studies for systolic blood pressure was I2 = 68% with no significant difference from baseline (-0.34 mmHg, 95% CI: -5.19 to 4.51, P = 0.89, Table 3: Analysis 3.15). The heterogeneity was due to one study [38], but removing this study did not change the non-significant results (1.76 mmHg; 95% CI: -2.61 to 6.13, P = 0.43, I2 = 42%). Neither was the diastolic blood pressure influenced by the DSME (-0.46 mmHg; 95% CI: -2.31 to 1.39, P = 0.63, I2 = 27%, Table 3: Analysis 3.16). At 12 months, two studies measured blood pressure [38,56]). There was no heterogeneity between the studies for systolic blood pressure (I2 = 0%). Although there was a small reduction in respect of systolic blood pressure, it was not statistically significant (3 mmHg; 95% CI: -7 to 2, P = 0.22, Table 3: Analysis 3.17). For diastolic BP, there was substantial heterogeneity of I2 = 70% (0.17; 95% CI: -4.46 to 4.80, P = 0.94). Neither of the two studies reported a significant difference between the intervention group and control group for diastolic blood pressure.

Seven studies including 1161 participants assessed total cholesterol and triglycerides at six months [34,38,44-47,49] with no significant differences (Total cholesterol -0.06 mmol/l; 95% CI: -0.23 to 0.12, P = 0.54, I2 = 4%, Table 3: Analysis 3.18; Triglycerides: -0.05 mmol/l; 95% CI: -0.19 to 0.08, P = 0.45, I2 = 37%, Table 3: Analysis 3.19). At 12 months, four studies [34,38,41,56] involving 657 patients displayed no statistically significant differences between groups (0.07 mmol/l, 95% CI: -0.09 to 0.24, P = 0.38, I2 = 0%, Table 3: Analysis 3.20) for total cholesterol. With regard to triglyceride levels, heterogeneity was high (I2 = 80%), though there were no differences between the groups (0.03 mmol/l, 95% CI: -0.42 to 0.48, P = 0.88, Table 3: Analysis 3.21). The high heterogeneity was caused by one study [41] which was the only study that reported a reduction in triglyceride levels in intervention group at 12 months (-0.52 mmol/l, 95% CI: -0.82 to -0.22, P = 0.0006). When this study [41] was removed from the meta-analysis, the heterogeneity among the remaining three studies reduced to 0% but still there were no statistically significant differences (0.16 mmol/l, 95% CI: -0.06 to 0.39, P = 0.15, Table 3: Analysis 3.21). High density lipoproteins (HDL) and low density lipoproteins (LDL) were assessed at six months by six studies [38,44-47,49] with a total of 932 participants. HDL analysis revealed no heterogeneity among the groups (I2 = 0%) and also a non-significant decrease in the HDL (-0.01 mmol/l, 95% CI: -0.05 to 0.03, P = 0.75, Table 3: Analysis 3.22) among the intervention group. LDL analysis also showed a non-significant reduction in the intervention group (-0.05 mmol/l, 95% CI: -0.2 to 0.1, P = 0.54, I2 = 37%, Table 3: Analysis 3.23).

Two studies [37,38] with 390 participants measured change in treatment satisfaction at six months. There was no heterogeneity between the studies (I2 = 0%), and they found that the group education participants were significantly more satisfied with their treatment (SMD 0.65; 95% CI: 0.44 to 0.85, P < 0.00001, Table 3: Analysis 3.24). At 12 months, three studies including 484 participants reported on treatment satisfaction [37,38,41]. The intervention group was significantly more satisfied with their treatment than the control group (SMD 0.39; 95% CI: 0.21 to 0.57 P < 0.0001, I2 = 0%, Table 3: Analysis 3.25).

At the 12 month outcome assessment there had been a total of 17 deaths reported from four studies with a combined total of 700 participants [32,36,38,51]. There was low heterogeneity (I2 = 3%). One study reported more deaths in the control group [38], whereas two studies reported more deaths in the intervention groups [36,51]. One study [32] had the same number of deaths in the intervention group as in the control group. Overall there were nine deaths in the intervention group and eight deaths in the control group. At the five year follow-up, the Trento study [54] also reported three deaths in the intervention and five in the control group respectively. Participation in a DSME, therefore, was not shown to affect mortality rate (odds ratio 1.1, 95% CI: 0.37 to 3.29, P = 0.87, Table 3: Analysis 3.26).

Only one study [38] reported the changes in medication at the 14 months follow-up period. Twenty-four (16%) reduced medication in the intervention group compared to one (0.7%) in the control group. Ninety-five (63%) intervention group patients and 75 (53%) control group patients remained on the same dose. Thirty-one (21%) intervention group patients and 65 (46%) control group patients increased their diabetes medication. The study concluded that for every seven patients who participated in the programme, one patient could be expected to have reduced their diabetes medication by 14 months (95% CI: 5 to 11, p < 0.0001).

Two studies reported cost but did not carry out cost effectiveness analysis [34,50]. The first study [34] reported that the cost of providing the intervention (52 contact hours over 12 months) was US $384 per person and the second study [50] reported that the total direct and indirect costs of providing the intervention were US $2,510 per participant for the 24 months period. One study did a cost effectiveness analysis [53]. It was found that over the study period group care required 196 minutes and US $756.54 per patient, compared with 150 minutes and US $665.77 for the control patients. It was reported that an additional US $2.12 was spent per point gained in the quality of life score.

Only one study monitored the presence of diabetes complications and it reported no significant differences between the group education participants and controls in respect of diabetic retinopathy and foot ulcers at two years [52] but found that at four years follow-up [53], diabetic retinopathy had progressed more slowly amongst participants that had attended the DSME (P < 0.009). No adverse effects were reported for the group education participants or the controls.

### Subgroup analyses

Subgroup analyses were done on the studies that had 12 months data on glycated haemoglobin (Table 4). Due to heterogeneity the changes from baseline to 12 months was used, giving a difference of glycated haemoglobin at 12 months of -0.55 percentage points (95%CI: -0.75 to -0.35, P < 0.00001, I2 = 37%).

**Table 4:** **Sub group analysis of the studies that had 12 months data on glycated haemoglobin (changes from baseline to 12 months) with comparison for intervention (Int) and control (contr) groups and the heterogeneity (measured by I**^**2**^**) of the analyses**

**Analysis number / Outcome**	**Effect measure**	**N studies**	**N participants (Int/contr)**	**Difference (95%CI)**	**P-value**^**A**^	**Heterogeneity (I2)**
4.1 Ethnicity (Most non-Caucasian)	Mean Diff	2	208/202	-0.49 (-0.97 to -0.01)	0.046	0.0
4.2 Theoretical model stated:	Mean Diff	4	309/291	-0.43 (-0.79 to -0.08)	0.017	52.1
4.3 Educator stated: Diabetes specialist nurse only	Mean Diff	3	217/219	-0.72 (-1.01 to -0.43)	0.000	35.8
4.4 Educator stated: Dietician only	Mean Diff	2	168/159	-0.80 (-1.22 to -0.37)	0.000	15.2
4.5 Educator stated: Studies with more than 1 instructor	Mean Diff	6	365/375	-0.27 (-0.53 to -0.02)	0.038	0.0
4.6 Delivered as Primary care intervention	Mean Diff	6	564/572	-0.61 (-0.85 to -0.37)	0.000	48.6
4.7 Both groups glycated haemoglobin 7 % & above at baseline	Mean Diff	8	553/532	-0.45 (-0.69 to -0.20)	0.000	24.1
4.8 Studies with follow-up sessions/phone calls before 12 months	Mean Diff	2	78/85	-0.14 (-0.56 to 0.27)	0.500	0.0
4.9 Studies with follow-up sessions/phone calls after 12 months	Mean Diff	2	153/161	-0.61 (-0.95 to -0.27)	0.000	10.7
4.10 Delivery of education programme completed in 1-5 months	Mean Diff	4	359/339	-0.65 (-0.87 to -0.42)	0.000	0.0
4.11 Delivery of education programme completed in 6-10 months	Mean Diff	3	104/124	-0.69 (-1.34 to -0.03)	0.040	79.7
4.12 Delivery of education programme completed in 12 months	Mean Diff	4	287/290	-0.35 (-0.67 to -0.02)	0.035	0.0
4.13 First quartile hours of education programme (8 hours or less)	Mean Diff	3	202/212	-0.55 (-0.86 to -0.23)	0.001	11.2
4.14 Second quartile hours of education programme (9 to 12 hours)	Mean Diff	3	225/225	-0.39 (-0.81 to 0.03)	0.068	57.4
4.15 Third quartile hours of education programme (13 to 18 hours)	Mean Diff	2	71/54	-0.69 (-1.74 to 0.37)	0.201	66.2
4.16 Fourth quartile hours of education programme (19 to 52 hours)	Mean Diff	3	252/262	-0.73 (-1.08 to -0.38)	0.000	27.8
4.17 Attendance rate less than 70 %	Mean Diff	3	132/124	-0.22 (-0.60 to 0.15)	0.243	0.0
4.18 Number of participants in each group session between 5 & 10	Mean Diff	3	124/135	-0.17 (-0.50 to 0.16)	0.321	0.0
4.19 Number of participants in each group session between 14 & 18	Mean Diff	3	264/249	-0.67 (-1.06 to -0.27)	0.001	28.8
4.20 Family & friends included in group sessions	Mean Diff	3	194/201	-0.41 (-0.83 to 0.01)	0.053	0.0
4.21 Number of sessions (5 or less)	Mean Diff	4	244/258	-0.52 (-0.75 to -0.28)	0.000	0.0
4.22 Number of sessions (6 to 10)	Mean Diff	4	265/255	-0.76 (-1.04 to -0.48)	0.000	28.0
4.23 Number of sessions (11 or more)	Mean Diff	3	241/240	-0.42 (-0.82 to -0.02)	0.040	0.0

A. The P- value is calculated for the difference between the intervention and control group.

Limiting the analysis to studies with 8 hours or less of education provided resulted in little or no change in the main outcome (-0.55) [39,42,51].

Studies with mainly a non-Caucasian sample [34,36], reporting theoretical model (-0.43) [32,37,38,51,56], using a combination of different types of educators to deliver the intervention [32,34,36,39,48,51], baseline haemoglobin levels of 7% or higher (-0.45) [32,34,36-39,51,56], that included follow up (-0.14) [32,39], completed delivery of their education programmes in 12 months (-0.35) [34,36,48,51], provided 9 to 12 hours of education (-0.39) [32,38,48], had a family member or friend was invited to attend as participants (-0.41) [34,39,51] or had less than 6 (-0.52) [32,39,42,51] or more than 10 sessions (-0.42) [34,36,48] had less effect of the intervention than all the studies together.

Studies using diabetes specialist nurses as the only type of educator (-0.72) [37,41,42], dieticians only as educators (-0.80) [38,56], conducted in primary care settings (-0.61) [32,34,36,38,41,42], intervention lasting from one to five months (-0.65) [37-39,42] or 6 to 10 months (-0.69) [32,41,56], provided 19 to 52 hours of education (-0.73) [34,36,41], between 14 and 18 participants per group session (-0.67) [36,38,56] or between 6 and 10 sessions (-0.76) [37,38,41,56] had better effect of the intervention than all the studies together.

### Sensitivity analyses

The sensitivity analyses were also performed on the subsets of studies glycated haemoglobin at 12 months (Table 5). Only two studies were assessed as having low risk of bias [32,38] and these produced a non-significant effect (-0.42 percentage points, 95% CI: -1.00 to 0.17, P = 0.16, I2 = 77%, Table 5: Analysis 5.4). The seven studies with moderate risk of bias [36,37,39,41,42,48,51] gave a statistically significant difference of -0.55 percentage points (95% CI: -0.79 to -0.31, P < 0.00001, I2 = 30%, Table 5: Analysis 5.5). Two studies with high risk of bias [34,56] produced no heterogeneity (I2 = 0%) and a significantly high effect of -0.95 percentage points (95% CI: -1.55 to -0.35, P = 0.002, Table 5: Analysis 5.6). An analysis of the two low risk and seven moderate risk studies, gave a difference of -0.51 percentage points (95% CI: -0.72 to -0.29, P < 0.00001, I2 = 41%, Table 5: Analysis 5.7). These results indicate that despite having greater intervention effects, the high risk of bias studies did not influence the overall result substantially.

**Table 5:** **Sensitivity analysis of the studies that had 12 months data on glycated haemoglobin (changes from baseline to 12 months) with comparison for intervention (Int) and control (contr) groups and the heterogeneity (measured by I**^**2**^**) of the analyses**

**Analysis number / Outcome**	**Effect measure**	**N studies**	**N participants (Int/contr)**	**Difference (95%CI)**	**P-value**^**A**^	**Heterogeneity (I2)**
5.1 Non-translated publications	Mean Diff	10	630/630	-0.52 (-0.75 to -0.28)	0.000	41.0
5.2 Number of participants more than median of all studies with 12 months HbA1c data(104 total)	Mean Diff	6	568/576	-0.67 (-0.86 to -0.48)	0.000	18.4
5.3 Studies with any part of results recalculated (HbA1c 12-14 months)	Mean Diff	6	450/438	-0.54 (-0.78 to -0.31)	0.000	0.0
5.4 Study quality (Low risk of bias):glycated haemoglobin 12-14 months	Mean Diff	2	192/187	-0.42 (-1.00 to 0.17)	0.163	77.4
5.5 Study quality (Moderate risk of bias)	Mean Diff	7	428/436	-0.55 (-0.79 to -0.31)	0.000	29.7
5.6 Study quality (High risk of bias)	Mean Diff	2	130/130	-0.95 (-1.55 to -0.35)	0.002	0.0
5.7 Study quality (Low & Moderate risk of bias)	Mean Diff	9	620/623	-0.51 (-0.72 to -0.29)	0.000	41.3
5.8 Drop-out rate less than 10 %	Mean Diff	5	444/454	-0.79 (-0.97 to -0.61)	0.000	0.0

A. The P- value is calculated for the difference between the intervention and control group.

The studies that had some of their results recalculated to make them suitable for entering in a meta-analysis (-0.54) [34,36,37,39,42,48] had similar effect to the overall analysis. The studies that which had each a total number of participants more than the identified median of 104 participants (-0.67) [34,36,38,41,42,51] or dropout rates of less than 10% (-0.79) [34,38,41,42,56] had better effect.

## Discussion

In total 21 studies (26 publications, 2833 participants) of group-based, diabetes self-management education programmes for people with type 2 diabetes were included. It was found that these programme overall resulted in significant health outcomes like improved glycaemic control and increased diabetes knowledge, self-management skills and self-efficacy/empowerment.

### Limitations

As a large number of outcomes and subgroups have been analysed, there is a possibility of type II error. The methodological quality of studies included in the review was mainly assessed as moderate. However, unlike a drug/placebo trial, it is very difficult to provide allocation concealment and to blind the patients and providers for a group-based educational intervention. Several of the studies were conducted before analysis by intention-to-treat became the norm. It was not possible to carry out a meta-analysis on several of the main outcomes due to very high heterogeneity between studies, and/or too few studies reporting on the outcome. The studies were mostly carried out in different developed countries throughout Europe and the United States. The results of this review are therefore likely generalisable to adults with type 2 diabetes in many different developed countries and there is no evidence to suggest that group-based self-management strategies would not be suitable for developing countries as long as the DSME is delivered in a familiar language and is sensitive to the culture of the population.

Although with some variation, the patients included in the studies were surprisingly similar. They were on average 60 years old, 40% were male, they were diagnosed seven years ago, four out of five used medications, and they had a mean HbA1c level of 8.23%. This indicates that most participants had a long history of living with type 2 diabetes. Thus, using the results from this systematic review and from most of the single studies included must be seen in relationship to the characteristics of those participating. For clinical practice, this indicates that the current knowledge of the effect of group based DSME is among a population with a rather long history of living with type 2 diabetes. It is therefore possible that the findings could be different in other populations. Furthermore, as with all clinical trials, it is possible that patients who participated in the studies may not be truly representative of the local adult population with type 2 diabetes, as people who volunteer to take part in clinical trials tend to be a more committed and motivated and may generally receive more attention when participating in a clinical trial. Although having motivated participants will not affect differences between the two groups as both the intervention and control group are part of the motivated subgroup, it may affect the generalisability of the results to DSME’s delivered as routine treatment.

### Findings

The meta-analysis of HbA1c at all time points from six months to five years showed a significant improvement in the intervention group that received group based DSME. The same was the case for fasting blood glucose. The improvement in glycaemic control found in this study is higher than the effect seen in previous studies analysing educational and behavioural interventions in type 2 diabetes [17,27] but lower than the effect seen in the study comparing the effectiveness of psychological specialists and general clinicians in the delivery of psychological educational programmes [18]. Furthermore, it is similar to the first review of group based DSME by Deakin et.al [28]. In conclusion, it can be said that group based DSME in general helps improve the participants’ glycaemic control.

There were clear indications in this review that studies with more numbers of hours of education (19 to 52 hours), spread over 6 to 10 months or with 6 to 10 sessions tended to do better. Other reviews with different inclusion criteria have reported that it appeared as though features of intervention and trial design such as length of follow-up and duration of intervention may not be as important as the number of sessions provided [18]. Yet another review reported that positive effects may be attributable to longer-term interventions with a shorter duration between the end of the intervention and the follow-up evaluation point [22]. On the other hand, it has been found that self-care management interventions may have a higher effect if the programme is compact with sessions closely grouped together [17]. Although these findings gives some directions for diabetes educators and other healthcare providers engaged in group based DSME, the magnitude of the education programme still remains as one area that needs further investigation.

It has been suggested that education delivered by a team of educators, with some degree of reinforcement of that education made at additional points of contact, may provide the best opportunity for improvements in patient outcomes [22]. In the present study, based on those studies that reported HbA1C at 12 months, it was indicated that the largest effect was seen when having a dietician as the only educator, although this was only done in two studies. The studies with only a diabetes specialist nurse as educator also tended to do better than the studies with a group of different educators. However, due to few studies no clear conclusion can be drawn whether having one person delivering the intervention is best. In addition, studies with one person delivering the intervention could measure that person's ability and engagement more than the actual content and quality of the intervention. Thus, transferring the same program delivered by one person in one study to another setting and person might not give the same results. The implications for those working clinically in this field is that if the program is delivered by only one person, the clinical, pedagogical and personal qualities of this person should be of the highest standards.

The five studies reporting having used a theoretical model in the development of their DSME showed less effect of the intervention. This indicates that having a theoretical model underpinning the program is not needed to achieve better results. Although it might be that other studies used a theoretical model but did not report this, it still raises the question about the usefulness of such models. One possible explanation is that clinical experience with the input from participants might provide an intervention that has better effect. This should encourage diabetes educators and other healthcare providers engaged in group based DSME to include participants in the planning, carrying out and evaluation of the program.

## Conclusion

Based on current evidence, there are indications that interventions delivered by a single educator, delivered in less than ten months, with more than 12 hours and between 6 and 10 sessions give the best results but more research is needed to confirm this. In general it can be concluded that group-based DSME in people with type 2 diabetes results in improvements in clinical, lifestyle and psychosocial outcomes.

## Appendix A. Example of search strategy

### MEDLINE

1. exp Patient Education/

2. exp Self Care/

3. exp Behavior Therapy/

4. exp Group Processes/

5. exp Psychotherapy, Group/

6. exp Self-Help Groups/

7. (empowerment or self care).tw,ot.

8. (patient$ adj6 education$).tw,ot.

9. behavio?r$ therap$.tw,ot.

10. (educational adj6 program$).tw,ot.

11. (self adj6 (care or efficac$ or help group$)).tw,ot.

12. (group$ adj6 (method$ or management$ or based or process$ or psychotherap$)).tw,ot.

13. (physical adj6 (training$ or education$)).tw,ot.

14. or/1-13

15. exp Diabetes Mellitus, Type 2/

16. exp Diabetes Complications/

17. (MODY or NIDDM or T2DM).tw,ot.

18. (non insulin$ depend$ or noninsulin$ depend$ or noninsulin?depend$ or non insulin?depend).tw,ot.

19. ((typ$ 2 or typ$ II) adj3 diabet$).tw,ot.

20. ((keto?resist$ or non?keto$) adj6 diabet$).tw,ot.

21. ((late or adult$ or matur$ or slow or stabl$) adj3 onset).mp. and diabet$.tw,ot.

22. or/15-21

23. exp Diabetes Insipidus/

24. diabet$ insipidus.tw,ot.

25. 23 or 24

26. 22 not 25

27. Meta-analysis.pt.

28. exp Technology Assessment, Biomedical/

29. exp Meta-analysis/

30. exp Meta-analysis as topic/

31. hta.tw,ot.

32. (health technology adj6 assessment$).tw,ot.

33. (meta analy$ or metaanaly$ or meta?analy$).tw,ot.

34. ((review$ or search$) adj10 (literature$ or medical database$ or medline or pubmed or embase or cochrane or cinahl or psycinfo or psyclit or healthstar or biosis or current content$ or systemat$)).tw,ot.

35. or/27-34

36. randomized controlled trial.pt.

37. controlled clinical trial.pt.

38. randomi?ed.ab.

39. placebo.ab.

40. clinical trials as topic.sh.

41. randomly.ab.

42. trial.ti.

43. or/36-42

44. 35 or 43

45. (comment or editorial or historical-article).pt.

46. 44 not 45

47. 14 and 26 and 46

## Competing interest

The authors declare that they have no competing interests.

## Authors' contributions

AS development of new protocol, searching for trials, quality assessment of trials, data extraction, data analysis, review development and drafted the manuscript. LØR searching for trials, quality assessment of trials, data extraction, data analysis. ML quality assessment of trials, data extraction, data analysis. MBR Searching for trials, data extraction. AF searching for trials, quality assessment of trials. All authors read and approved the final manuscript.

## Pre-publication history

The pre-publication history for this paper can be accessed here:

http://www.biomedcentral.com/1472-6963/12/213/prepub
